# Modelling Polyphenol Extraction through Ultrasound-Assisted Extraction by Machine Learning in *Olea europaea* Leaves

**DOI:** 10.3390/foods12244483

**Published:** 2023-12-14

**Authors:** Raquel Rodríguez-Fernández, Ángela Fernández-Gómez, Juan C. Mejuto, Gonzalo Astray

**Affiliations:** Universidade de Vigo, Departamento de Química Física, Facultade de Ciencias, 32004 Ourense, Spain; raquel.rodriguez.fernandez@uvigo.gal (R.R.-F.); angela.fernandez.gomez@alumnos.uvigo.gal (Á.F.-G.); xmejuto@uvigo.es (J.C.M.)

**Keywords:** olive leaves, ultrasound-assisted extraction, extract yield, TPC, machine learning, random forest, support vector machine, artificial neural network

## Abstract

The study of the phenolic compounds present in olive leaves (*Olea europaea*) is of great interest due to their health benefits. In this research, different machine learning algorithms such as RF, SVM, and ANN, with temperature, time, and volume as input variables, were developed to model the extract yield and the total phenolic content (TPC) from experimental data reported in the literature. In terms of extract yield, the neural network-based ANN_Z-L_ model presents the lowest root mean square error (RMSE) value in the validation phase (9.44 mg/g DL), which corresponds with a mean absolute percentage error (MAPE) of 3.7%. On the other hand, the best model to determine the TPC value was the neural network-based model ANN_R_, with an RMSE of 0.89 mg GAE/g DL in the validation phase (MAPE of 2.9%). Both models obtain, for the test phase, MAPE values of 4.9 and 3.5%, respectively. This affirms that ANN models would be good modelling tools to determine the extract yield and TPC value of the ultrasound-assisted extraction (UAE) process of olive leaves under different temperatures, times, and solvents.

## 1. Introduction

Olive leaves (*Olea europaea*) are an interesting waste by-product of the olive oil industry [1]. Generally, they are acquired during olive harvesting or fabrication operations [2]. In the European Union (EU-28), oil mills generate 9.6 million tons/year of by-products that can be recovered, such as olive pomace, stone, and olive tree pruning biomass, which represents an additional 11.8 million tons [3]. Olive oil is the main lipid component of the Mediterranean diet [4]. As reported by Ben Hmida et al. (2022) [5], olive oil production and *Olea europaea* cultivation are significant and ancestral agricultural activities in Mediterranean countries. The EU produces around 67% of the world’s total olive oil (mainly in the Mediterranean EU countries, representing about 4 million hectares [6].

The olive plant has phenolic compounds that present beneficial health effects [7] and, as reported de Bock et al. (2013) [7], could possess antioxidant or anti-inflammatory properties, among others. Polyphenols are present principally in the tree’s leaves and drupes [8]. The most ample phenolic compound in olive leaves is oleuropein [9]. As Martín García (2001) [10] reports in his Ph.D. thesis, the chemical composition of olive leaves, and therefore also their nutritional value, depends on a large number of factors such as climatic conditions, the variety of olive, the age of the plantation, or the harvest season. In fact, in the literature, studies can be found such as that by Cavalheiro et al. (2015) [11], in which the olive leaves of different varieties from Brazil are analysed according to moisture, ash, proteins, lipids, etc., or the research carried out by Ibrahim et al. (2016) [12], where different chemical compositions of whole and boiled leaves were studied.

On the other hand, Martín García (2001) [10] also reports that olive leaves generally have greater digestibility and a higher nutritional value than other by-products of oil extraction, but with a general observation that olive leaves have a low protein value.

Taking into account the trend to find abundant, renewable, and cheap sources of polyphenols [13], it can be understood that the characteristics that are present in olive leaves, together with the benefits that biophenols present, have given rise to a growing interest in their use in different industrial applications in the food supplement or pharmaceutical fields [2]. Nevertheless, the polyphenol profile in olive leaves is susceptible to several abiotic and biotic factors, such as the geographical zone, leaves, and so on [14]. On the other hand, there are certain important parameters that influence the extraction processes: the type and composition of the solvent, the extraction time, or the extraction temperature, among others [13]. Consequently, it is necessary to design and optimize the extraction methods for each polyphenol source [13].

Ultrasound-assisted extraction (UAE), as reported by Yerena-Prieto et al. (2022) [15], is a methodology based on cavitation, a phenomenon that causes the disruption of cell walls and having the effect that the release of target compounds [16]. In terms of the sound emitter devices (one of the parts of the core of ultrasonic equipment), two different types of devices can be used: an ultrasonic bath and a sonotrode (ultrasonic probe) [17]. As reported by Chahardoli et al. (2020) [18], UAE is a non-complex and reasonably priced method that can be applied at large scales for industrial and commercial purposes, can afford an improvement in mass transfer, provides an option for high-temperature procedures and a reduction in extraction time, and other economical or environmental benefits [19,20,21,22].

The high efficiency is caused by to the acoustic cavitation effect produced by the formation and implosion of microbubbles generated by ultrasonic waves [23].

The optimization and prediction of different properties in extraction methodologies can be approximated by traditional modelling methods such as response surface methodology (RSM) [13,24] or kinetic modelling [25]. Machine learning is an alternative to traditional methods; examples of this are the models based on random forest (RF), support vector machine (SVM), and artificial neural networks (ANNs).

According to Tian et al. (2017) [26], Breiman introduced RF for the first time in 2001 [27]. It is a classification and regression method that relies on statistical learning theory [26]. RF combines many prediction trees, where each tree is based on the values of an independently sampled random vector that presents the same distribution in all of the forest’s trees [27]. In classification, the RF output corresponds to the class selected by a majority vote of all individual trees [26]; in regression, the predicted value is determined by averaging over all the trees [28]. RF can be utilized in different research fields, such as in agricultural science to detect nitrogen saturation [29], in environmental sciences to evaluated the exposure to particular matter in urban areas [30], and in food technology for quality control [31] and to detect food adulteration [32].

The next model, SVM, was first proposed, according to Waleed et al. (2020) [33], by Cortes and Vapnik in 1995 [34]. SVM is a supervised non-parametric statistical learning method; hence, it is not necessary to know the data distribution beforehand [35,36]. The operation is based on building a hyperplane to separate the data into different classes [35], while minimizing the classification error, through geometric margin maximization between classes [33]. SVM can be applied in different research areas, such as in chemistry to predict the toxicity of different compounds [37,38], in hydrology to determine water quality for drinking purposes [39], and in food technology to study plasticizers in extra virgin olive oil [40] or to classify Greek olive oils [41], among others.

Finally, the ANN technique is based on a computational system inspired by biological neural networks, which is composed of an input, an intermediate, and an output [42]. These networks consist of a set of artificial neurons that receive a series of signals (called inputs) that, once processed, if these signals exceed a threshold value, will be transmitted to another neuron [42]. According to Silva et al. (2015) [43], the ANN algorithm offers several advantages over conventional methods, such as its ability to fill in missing data, its non-linearity, insensitivity to noise or high parallelism, among others [44]. ANNs have versatile applications across various fields of research, like in food technology for honey classification [45] or wine authentication [46], in chemistry to determine the electrical percolation of AOT-based microemulsions [47], and so on.

To summarize, in this study, different machine learning methods, (i) RF, (ii) SVM, and (iii) ANN, were applied to model the extract yield (mg/g DL)—DL corresponds to dried leaf—and the total phenolic content (TPC) (mg GAE/g DL) using experimental data obtained from the literature [25].

## 2. Materials and Methods

### 2.1. Experimental Data

The data used in this research were obtained from the experimental work carried out by Şahin (2019) [25]. According to Şahin (2019) [25], olive leaves were obtained from the Mediterranean region of Kaş (Turkey) during November in 2013, which is when the leaves have the highest content of phenolic content [48]. The leaves were dried under ambient conditions without exposure to light, and then stored in plastic bags in the absence of light until the time of grinding for extraction [25].

According to Şahin (2019) [25], UAE was performed using different solvent concentration ratios (aqueous solutions (*v/v*) at 50% of ethanol or methanol, and water, EtOH, or MeOH pure solutions) under a specific temperature (30 to 80 °C in steps of 10 °C for water; 30, 40, and 50 °C for EtOH, MeOH, and their aqueous solutions) and time range (20 to 60 min in steps of 10 min), with ninety being the total number of experimental cases.

The method developed by Malik and Bradford (2006) [49] was used by Şahin (2019) [25] to quantify the total polyphenolic content (TPC) present in the extract samples. A second-order kinetic model was used by Şahin (2019) [25] to carry out the kinetic description of the extraction.

### 2.2. Machine Learning Approximations Developed

The database was divided into three groups: training (50%), validation (30%), and testing (20%). The training dataset, as the name suggests, was used to train the model, the validation group was used to find the best model, and the test group was retained to check if the model fit well with other external data. Temperature (°C), time (min), and volume (*v/v*) for each solvent type were used as input variables.

**Figure 1:**
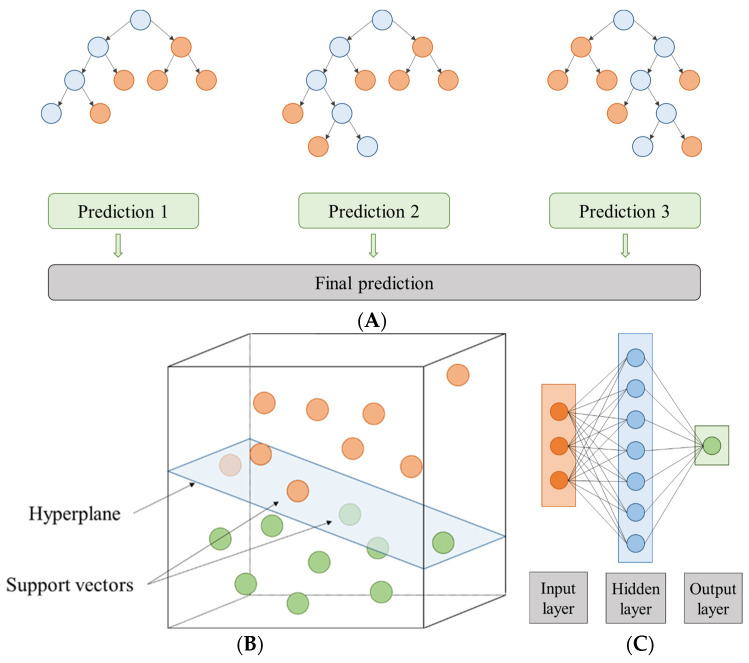
Scheme of (**A**) a random forest made up of three individual prediction trees, (**B**) a hyperplane in R3, and (**C**) an artificial neural network with a topology of 3-7-1.

The first approach developed was random forest. The RF algorithm involves the random selection of samples from the original training dataset [50]. Its operation is based on a general classifier that is composed of several individual decision trees [51] (Figure 1A). Each of these individual classifiers is generated through a random vector that is taken independently from the input vector [51], and then their predictions are averaged [50]. This method has multiple benefits because the bootstrapping process decreases model variance without a bias increment [50,52]. It is also fast to analyse and is not vulnerable to overtraining and noise compared to other boosting-based techniques [50,52]. In this research, to find the best RF model, the following hyperparameters were tested: number of trees (in the range 1–100 using 99 steps), maximum depth (in the range 1–100 using 99 steps), attribute selection criteria (criterion least square), and pre-pruning (true or false). In addition, normalized RF models were also performed to fit the values to a specific range as the data had different units and scales. Two normalization methods were used: Z-transformation (indicated with subscript Z), which is a method of statistical normalization, and range transformation between −1 and 1 (indicated with subscript R). The normalization process was applied to the training group and, later, this normalization model was applied to the other data groups.

The SVM method is a classifier focused on splitting two linearly separable classes by searching for an optimal hyperplane [50,53] (Figure 1B). According to [50], to find the best hyperplane it is necessary to maximize the distance between the closest training cases (support vectors) and the separating hyperplane. To solve the SVM multiple classification problem, as reported by Wang et al. (2020) [54], a combination of multiple binary classifiers is mainly used [55]. In this study, epsilon-SVR and nu-SVR were used to classify SVM types. In addition, C and gamma parameters were chosen to train the data. These parameters can be set according to the guide of Hsu et al. (2003) [56]. However, these working ranges were extended to improve model fits: the range of gamma values was approximately 9.5 × 10^−7^ to 256 with 28 steps and the range of C values was approximately 9.8 × 10^−4^ to 1,048,576 with 30 steps. The SVM library, proposed by [57], known as LibSVM, was used [58]. The two normalization methods, mentioned above, were also used: the Z-transformation and the range transformation between −1 and 1. In addition, the models were also applied in linear scale and logarithmic scale (indicated with subscript L) for gamma and C.

ANN modelling is a simplified imitation of the human brain that it is composed of parallel processing units like the neurons present in living creatures [59]. In this study, the multilayer perceptron (MLP) was composed of input, intermediate, and output layers (each of these layers with a certain number of neurons) (Figure 1C). The input nodes depend on the experimental set, so in this case there were five (temperature, time, and volume % for each solvent type). In the intermediate layer, the neurons number was defined by 2n + 1, with “n” being the number of input variables. Moreover, the neural models were developed with different hyperparameter configurations: training cycles (between 1 and 524,288 using 19 steps) and decay (true or false). In addition, the two normalization methods, Z-transformation and range transformation between −1 and 1 scale, were carried out again on each of them, and linear scale and logarithmic scale were used for the training cycles.

General schemes for the RF, SVM, and ANN approaches that intended to model the extract yield and total phenolic content can be seen in Figure 1.

### 2.3. Best Model Selection

In this research, several statistical parameters were utilized to analyse the implemented machine learning models: (i) the root mean square error (RMSE), (ii) the mean absolute percentage error (MAPE in %), and (iii) the correlation coefficient (r); these were determined for the training, validation, and testing groups. The equations can be consulted in Naeem et al. (2021) [60]. The model with the lowest RMSE value was considered the best model for each approach.

### 2.4. Equipment and Software

The obtained data were collected using Microsoft Excel 2013 (Microsoft, Redmond, WA, USA). The different models were created with RapidMiner Studio Educational 9.10.001 version (RapidMiner an ALTAIR Company, Troy, MI, USA). The computational equipment used was an Intel^®^ Core™ i7-8700 CPU at 3.20 GHz with 64 GB of RAM and Windows 10 Pro. Figures were plotted with the SigmaPlot 13.0 (Systat Software Inc., San José, CA, USA).

## 3. Results and Discussion

In the following sections, corresponding to the Results and Discussion, the machine learning models selected based on the RMSE in the validation phase are detailed. Likewise, a discussion of the results is also carried out, comparing them with other research articles located in the literature.

### 3.1. Models to Yield Determination

Table 1 is shown below, presenting the models selected for each of the types of approximation carried out for the determination of yield and total polyphenol content.

For extract yield modelling, the first group of models developed was the random forest. Among this kind of approximation, the best RF model present an RMSE value of 13.42 mg/g DL in the validation phase that corresponds with a correlation coefficient of 0.962 (Table 1). The other two selected models developed (RF_R_ and RF_Z_) showed slightly higher RMSE values (13.63 and 14.06 mg/g DL, respectively). The mean absolute percentage error is practically the same in all three models and varies around the range 5.0 to 5.5%. The selected RF model offers better adjustments for the training phase than for the validation phase. This can be seen in the different values obtained for the statistics under study, showing a clear decrease in the RMSE and the MAPE (8.20 mg/g DL and 3.3%) and an increase in the correlation coefficient value (0.991).

The second group of models (six SVM) were developed at the linear scale (SVM, SVM_R_, and SVM_Z_) and the logarithmic scale (SVM_L_, SVM_R-L_, and SVM_Z-L_). The RMSE results obtained from the SVM models at the logarithmic scale (9.87, 10.53, and 11.02 mg/g DL for SVM_L_, SVM_R-L_, and SVM_Z-L_, respectively) are significantly lower than the result obtained at the linear scale (14.16, 37.88, and 37.68 mg/g DL for SVM, SVM_R_, and SVM_Z_, respectively). Considering these results, it can be concluded that the best SVM model, considering the lowest RMSE error for the validation phase, is the SVM_L_ model that presented an RMSE value of 9.87 mg/g DL (Table 1). These good adjustments in terms of RMSE are also reflected in the MAPE (3.9%) and in the correlation coefficient (0.979) obtained. These good adjustments are also obtained in the training, where the model presents very similar values (RMSE of 8.36 mg/g DL and correlation coefficient of 0.986) to those reported for the validation.

Finally, the last kind of model was the artificial neural network. In this case, different ANNs were developed at the linear scale (ANN, ANN_R_, ANN_Z_) and the logarithmic scale (ANN_L_, ANN_R-L_, ANN_Z-L_). The root mean square error values obtained by these models in the validation phase varied between 14.16 mg/g DL (ANN_L_) and 9.44 mg/g DL (ANN_Z-L_). The two un-normalized models (ANN and ANN_L_) are the models with the highest RMSE in this phase (12.97 and 14.16 mg/g DL, respectively). The best model, as previously stated, is the ANN_Z-L_ model, which presents a root mean square error of 9.44 mg/g DL for this phase, which corresponds to a MAPE of 3.7% (Table 1). The good behaviour in the validation phase is widely exceeded in the training phase, where the ANN_Z-L_ model presents a relative RMSE of 2.71 mg/g DL and a mean absolute percentage error of 0.9%.

Among the three selected models chosen by the lowest RMSE in the validation phase (Table 1), the one with the worst adjustments in the validation phase is the RF model (13.42 mg/g DL), followed by the SVM_L_ model (9.87 mg/g DL), which is very close to the best model, the ANN_Z-L_ model (9.44 mg/g DL), developed with Z-transform normalization and logarithmic scale (ANN_Z-L_). These three models improve, for the training group, the adjustments obtained in the validation phase (especially the ANN model: 9.44 vs. 2.71 mg/g DL). It seems clear that the ANN_Z-L_ model offers good results for both phases, training and validation, but it is necessary to verify its proper functioning in external data; for this, a previous group of data was reserved (test data). It can be seen that the ANN_Z-L_ model offers good results based on RMSE (12.82 mg/g DL) and MAPE (4.9%).

Despite the fact that the ANN_Z-L_ model offers a good fit for the test phase, it can be seen that one of the three previously selected models, the SVM_L_, offers better results for this phase. It can be seen that the root mean square error value is the lowest of the three selected models (5.95 mg/g DL); this also corresponds to the MAPE (2.6%).

Based on these results, it can be concluded that although the ANN_Z-L_ model is, within the three selected models, the one that presents the lowest RMSE in the validation phase, and works correctly in the external validation phase, the SVM_L_ model, whose statistics in the validation are very close to those obtained by the ANN_Z-L_ model, is also a good performance modelling method due to the low error obtained in the test phase.

**Figure 2:**
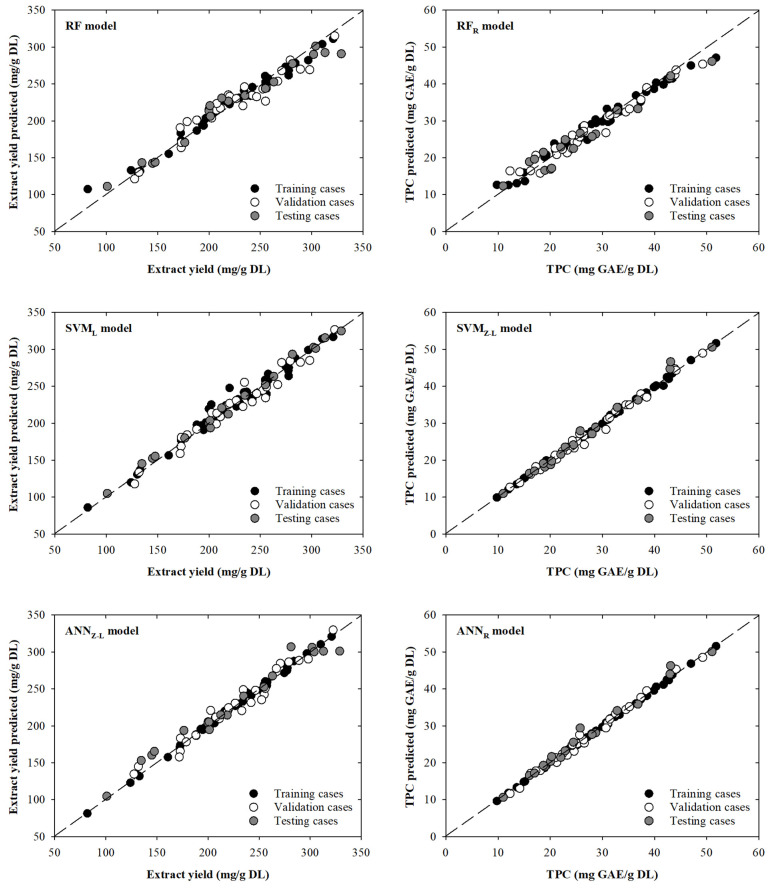
Scatter plots showing the real and modelled values of extract yield (**left**) and TPC (**right**) for the selected models. The black dashed line is the line with slope one.

The RF model intended to model extract yield showed little dispersion between the experimental and modelled data. However, it can be seen that some cases move away from the line with slope one. For example, one training experimental case (81.9 mg/g DL) presents a significant deviation from the dashed line: an overestimation of 31.1% that corresponds with a modelled value of 107.4 mg/g DL. Additionally, another case (328.8 mg/g DL) in the testing phase presents a slightly deviation from the line with slope one, being modelled as 290.8 mg/g DL, which implies an underestimation of 11.6%. Based on these results, it can be observed that dispersion is mainly centred on extreme values of the extract yield values. This behaviour is not the case for the SVM_L_ model, where dispersion is mainly concentrated in the middle–upper part of the graph. For instance, in one training case (220.0 mg/g DL), a value of 247.7 mg/g DL is modelled, resulting in an overestimation of 12.6%. The remaining cases in the training, validation, or testing phases are close to the line with slope one. This is clear in the test data that present better fits (RMSE = 5.95 mg/g DL) compared to the fits presented by the previous RF model (RMSE = 13.89 mg/g DL). On the other hand, as previously seen in the results shown in Table 1, the ANN_Z-L_ model presents the lowest dispersion for the TVZ datasets. In fact, the model presents a MAPE value of 2.6% for all the data. Even so, in the upper part of the graph, there are two testing cases (281.2 and 328.8 mg/g DL) that are far from the dashed line with modelled values of 307.0 and 301.1 mg/g DL, resulting in an overestimation of 9.2% and an underestimation of 8.4%, respectively. Although these points are the most attractive to the human eye, there are a series of points towards the lower–middle zone of the graph that present a greater overestimation between 13.6% and 9.8%. All these behaviours are reflected in Figure 2, where the scatter plots between the real value and those modelled by each of the selected models are shown. The points, for the extract yield models, are close, in general, to the black dashed line (line with slope one).

### 3.2. Models for Total Phenolic Content Determination

The second group of models selected to model the TPC are shown in Table 2. The first approach was, again, a group of three random forests. In this sense, the best random forest model (RF_R_) presented an RMSE value of 1.93 mg GAE/g DL for the validation phase that corresponds with a correlation coefficient of 0.982. The other two models developed (RF and RF_Z_) showed similar RMSE values with minimum differences between them (2.00 and 1.94 mg GAE/g DL, respectively). Analysing the relative error, it remains unchanged across the three models, varying around 6.5% to 6.8%. The RF_R_ model exhibits better adjustments during the training phase as opposed to the validation phase. These perceptible differences can be seen in the statistical parameters analysed: the RMSE and MAPE values slightly decreased (1.51 mg GAE/g DL and 4.6%), with an increase in the correlation coefficient value (0.990).

In addition to the RF models, a second group of six SVM models was also developed. The RMSE values obtained, 0.99, 0.95, and 0.93 mg GAE/g DL for SVM_L_, SVM_R-L_, and SVM_Z-L_, respectively, were significantly lower than for SVM, SVM_R_, and SVM_Z_ (1.89, 7.19, and 7.35 mg GAE/g DL, respectively). Based on these results, it can be said that the SVM_Z-L_ was the best model, obtaining an RMSE value of 0.93 mg GAE/g DL during the validation phase with a corresponding correlation coefficient of 0.995 and mean absolute percentage error of 2.7% (Table 2). Similar to the RF models, better fits were observed in the training phase than in the validation phase, with a decrease in RMSE and MAPE values (0.41 mg GAE/g DL and 0.8%, respectively) and an increase in the correlation coefficient value (0.999).

The final type of model included a set of six ANNs similar to the ANNs developed for extract yield. The un-normalized models in both scales (ANN and ANN_L_) showed slightly higher values of RMSE (1.52 and 1.70 mg GAE/g DL, respectively) compared to the normalized models (between 0.89 and 1.00 mg GAE/g DL, respectively). The best model was ANN_R_, which presented a root mean square error of 0.89 mg GAE/g DL in the validation phase that corresponds with a MAPE value of 2.9% and a correlation coefficient of 0.996 (Table 2). Consistent with previous findings, it can be observed that in the training phase the model shows better adjustment compared to the validation phase. In this case, the ANN_R_ model presents a descent in both RMSE and MAPE (0.28 mg GAE/g DL and 0.9%, respectively).

Considering the three selected models based on the lowest RMSE during validation, the best model is ANN_R_ (0.89 mg GAE/g DL), followed by SVM_Z-L_ (0.93 mg GAE/g DL) and the RF_R_ model (1.93 mg GAE/g DL). The correlation coefficients for all three models are high, greater than 0.980, and the relative errors are generally low (under 3.0%), except for the RF model, which exceeds 6.0%. All these models also show an improvement in the adjustment parameters for the training phase, especially the ANN_R_ model, which improved from 0.89 mg GAE/g DL to 0.28 mg GAE/g DL. While the ANN_R_ model appears to perform well in both phases, it is necessary to verify its performance using test data. In the testing phase, the ANN_R_ model offers good results (1.35 mg GAE/g DL of RMSE), but it fails to achieve the best result. In this case, the SVM_Z-L_ model performs better during testing with an RMSE of 1.23 mg GAE/g DL, a similarly high correlation coefficient (0.995 vs. 0.993), and a low MAPE value (3.1 vs. 3.5%). However, it can be considered that the differences between these two models, ANN_R_ and SVM_Z-L_, are minimal.

Based on all the above, it can be said that the results show that ANN_R_ has the lowest RMSE in the validation phase and works well in external validation. However, SVM_Z-L_ has similar statistics and performs well in the test phase too, which makes both models good modelling methods.

Analysing the scatter plots presented in Figure 2, it can be seen that the RF_R_ model designed to determine the TPC provides lower modelled values in the upper-right zone of Figure 2. Three specific cases of training, validation, and testing (51.8, 49.2, and 51.0 mg GAE/g DL), which decrease away from the dashed line (47.1, 45.4, and 46.1 mg GAE/g DL), present underestimations of 9.0, 7.8, and 9.6%. On the other hand, it can be seen that for the SVM_Z-L_ and ANN_R_ models, the dispersion around the trend line is smaller than for the RF_R_ model. For the SVM_Z-L_ and ANN_R_ models, two testing cases can be highlighted (25.8 and 43.1 mg GAE/g DL), with overestimations of 8.4 and 8.3%, and 14.2 and 7.4%, respectively, for each model. By comparing the graphs and the statistical parameters obtained, it can be concluded that not only can the ANN_R_ model be used, but also the SVM_Z-L_ model could be used to model the variables of interest. This can be corroborated using the MAPE value for the total dataset (1.8%).

The results obtained in this research can be considered good for all the selected models. This statement is based on the good results obtained in all three phases where the selected models present MAPE values between 1.8 and 6.1%, which means that the percentage errors obtained by these models can be considered acceptable. The results obtained in this research can be compared with others reported in the literature.

In this sense, Alrugaibah et al. (2023) [61] carried out the extraction of different phenolic compounds, using deep eutectic solvents, by means of neural networks. The authors used four input variables and were able to obtain RMSE values between 0.04 and 0.16 mg/g [61]. Although the RMSE values are lower than those obtained in this research, it is necessary to indicate that the ranges studied for each of the phenolic compounds is lower [61] than the range studied in this research for the total extract yield. İlbay et al. (2014) [62] developed response surface methodology (RSM) and artificial neural network approaches to model and optimize the extraction process. In that work, the range of TPC (21.56 to 47.58 mg GAE/g DL) is similar to the range analysed in this research (9.79 to 51.78 mg GAE/g DL). In terms of RMSE, it can be observed that the ANN model presents a lower value (1.13 mg GAE/g DM) compared to the RSM (1.85 mg GAE/g DM) [62]. A similar work using response surface methodology was carried out by Goldsmith et al. (2014) [63] using temperature, time, and sample-to-solvent ratio as input variables, obtaining a model that can predict the TPC with an RMSE of 3.79 mg GAE/g for experimental values around 20 to 40 mg GAE/g. In the present study, the selected ANN model shows, for the total phases, an RMSE of 0.80 mg GAE/g DL, which indicates that the ANN model would work in a similar, or better, way than the models reported by İlbay et al. (2014) [62] and Goldsmith et al. (2014) [63].

More examples have also been found in the specialized literature; in this sense, Şahin et al. (2017) [64] developed RSM and ANN models to model and optimize TPC and oleuropein yields in olive leaf obtained by the SFMAE method (solvent-free microwave-assisted extraction). After an analysis of variance (ANOVA), the ANN model provides better results for TPC and oleuropein amount than the RSM model.

## 4. Conclusions

In this research, three supervised machine learning approaches, random forest, support vector machine, and artificial neural network, were developed to model the extract yield and the total phenolic content extracted from *Olea europaea* leaves through ultrasound-assisted extraction. According to the results, it can be seen that the ANN models showed better performance in accuracy and generalization capability than the other models developed. Data normalization (Z-transformation and range transformation) was also identified as an important factor to improve the effectiveness of the ANN. In this sense, the best selected neural network models present Z-transformation for the extract yield ANN model and range transformation for the total phenolic content ANN model. These two models provide good results in all phases, showing, for the querying phase, MAPEs of 4.9 and 3.5%, respectively. These findings suggest that ANN models using an appropriate normalization technique can be promising tools to model the extraction yield and TPC value. However, more research could be necessary to improve these results and their applicability in the pharmaceutical, food, or chemical industries.

Finally, it is necessary to emphasize that, with the increasing awareness of environmental issues and the growing need to reduce time, economic/material/personal costs, and the use of toxic products, the combination of these two methods, UAE and machine learning, will be an area of great interest among researchers that could lead to an improvement in the specialized industry.

## Figures and Tables

**Table 1:** Adjustments for the selected algorithms to model the extract yield. RMSE corresponds to the root mean square error (mg/g), MAPE is the mean absolute percentage error (in %), and r corresponds to the correlation coefficient.

	Training			Validation			Test		
Model	RMSE	MAPE	r	RMSE	MAPE	r	RMSE	MAPE	r
RF	8.20	3.3	0.991	13.42	5.0	0.962	13.89	4.9	0.983
SVM_L_	8.36	2.9	0.986	9.87	3.9	0.979	5.95	2.6	0.997
ANN_Z-L_	2.71	0.9	0.999	9.44	3.7	0.980	12.82	4.9	0.985

**Table 2:** Adjustments for the selected machine learning algorithms to model the total phenolic content. RMSE corresponds to the root mean square error (mg GAE/g DL), MAPE is the mean absolute percentage error (in %), and r corresponds to the correlation coefficient.

	Training			Validation			Test		
Model	RMSE	MAPE	r	RMSE	MAPE	r	RMSE	MAPE	r
RF_R_	1.51	4.6	0.990	1.93	6.5	0.982	2.47	9.4	0.978
SVM_Z-L_	0.41	0.8	0.999	0.93	2.7	0.995	1.23	3.1	0.995
ANN_R_	0.28	0.9	1.000	0.89	2.9	0.996	1.35	3.5	0.993

## Data Availability

Data are contained within the article.
